# Maximal exercise test is a useful method for physical capacity and oxygen consumption determination in streptozotocin-diabetic rats

**DOI:** 10.1186/1475-2840-6-38

**Published:** 2007-12-13

**Authors:** Bruno Rodrigues, Diego M Figueroa, Cristiano T Mostarda, Marcelo V Heeren, Maria-Cláudia Irigoyen, Kátia De Angelis

**Affiliations:** 1 Hypertension Unit, Heart Institute (InCor), Medical School, University of Sao Paulo, Sao Paulo-Sao Paulo, Brazil; 2 Human Movement Laboratory – Sao Judas Tadeu University, Sao Paulo-Sao Paulo, Brazil

## Abstract

**Background:**

The aim of the present study was to investigate the relationship between speed during maximum exercise test (ET) and oxygen consumption (VO_2_) in control and STZ-diabetic rats, in order to provide a useful method to determine exercise capacity and prescription in researches involving STZ-diabetic rats.

**Methods:**

Male Wistar rats were divided into two groups: control (CG, n = 10) and diabetic (DG, n = 8). The animals were submitted to ET on treadmill with simultaneous gas analysis through open respirometry system. ET and VO_2 _were assessed 60 days after diabetes induction (STZ, 50 mg/Kg).

**Results:**

VO_2 _maximum was reduced in STZ-diabetic rats (72.5 ± 1 mL/Kg/min^-1^) compared to CG rats (81.1 ± 1 mL/Kg/min^-1^). There were positive correlations between ET speed and VO_2 _(r = 0.87 for CG and r = 0.8 for DG), as well as between ET speed and VO_2 _reserve (r = 0.77 for CG and r = 0.7 for DG). Positive correlations were also obtained between measured VO_2 _and VO_2 _predicted values (r = 0.81 for CG and r = 0.75 for DG) by linear regression equations to CG (VO_2 _= 1.54 * ET speed + 52.34) and DG (VO_2 _= 1.16 * ET speed + 51.99). Moreover, we observed that 60% of ET speed corresponded to 72 and 75% of VO_2 _reserve for CG and DG, respectively. The maximum ET speed was also correlated with VO_2 _maximum for both groups (CG: r = 0.7 and DG: r = 0.7).

**Conclusion:**

These results suggest that: a) VO_2 _and VO_2 _reserve can be estimated using linear regression equations obtained from correlations with ET speed for each studied group; b) exercise training can be prescribed based on ET in control and diabetic-STZ rats; c) physical capacity can be determined by ET. Therefore, ET, which involves a relatively simple methodology and low cost, can be used as an indicator of cardio-respiratory capacity in future studies that investigate the physiological effect of acute or chronic exercise in control and STZ-diabetic male rats.

## Background

Diabetes is a great public health problem associated with high morbidity and mortality. Frequently, this condition is accompanied by other disorders, such as coronary heart disease, hypertension, dyslipidemia and obesity [1]. One consequence of this metabolic condition is the reduced exercise capacity that has been commonly observed in diabetic patients. This reduction is probably dependent on various physiological factors, such as neuromuscular activity, hemodynamics, and respiratory mechanics and oxygen consumption [2].

Maximum oxygen consumption (VO_2 _max) test is highly reproducible, and it is considered a "gold standard" for functional capacity assessment in athletes and sick individuals. This parameter expresses the functional health of the cardiovascular, pulmonary, and skeletal muscle systems [3]. In addition, it provides important prognostic information that is useful for identifying potential candidates for cardiac transplantation [3,4]. VO_2 _max test also provides objective data, which is helpful in formulating appropriate exercise prescription [5,6]. However, sometimes VO_2 _max measurements have limited use in animal experimentation because of the high cost of the gas analyzer equipment [7] and its time-consuming characteristic.

Maximum and submaximum exercise tests (ET) have been considered safe and well tolerated. This method implies simple measures of functional capacity, and it has been used in clinics, physical qualification programs, and cardiac rehabilitation programs [8,9]. The assessment of cardio-respiratory responses to acute and chronic exercise, using different methods (as used in humans), has been a common practice in the investigation of animal models.

In experimental practice, VO_2 _max measurement is a noninvasive tool that has great value in the study of functional capacity of animals. Therefore, maximum ET, in which animal runs to exhaustion on a graded load treadmill, has been used by our group as an alternative method of evaluation of physical capacity, and it is also used for physical training prescription in health and pathological conditions [6,10-16].

Streptozotocin (STZ)-induced diabetes has been largely used in the study of the role of exercise training in diabetic-induced cardiovascular and autonomic dysfunction [6,14,16]. However, the relationship between the speed of ET and VO_2_, as well as the accuracy of ET in estimating cardio-respiratory capacity remains unclear in STZ-diabetic rats. Therefore, the purpose of the present investigation was to evaluate the relationship between the speeds of ET and both VO_2 _and VO_2 _reserve (VO_2 _res) and to validate the equations to predict the VO_2 _and VO_2 _res based on speeds of ET in control and STZ-diabetic rats.

## Methods

Experiments were performed on 18 untrained adult male Wistar rats (200–300 g) from the Animal House of the University of Sao Paulo, Sao Paulo, Brazil. Animals received standard laboratory chow and water *ad libitum*, were housed in collective polycarbonate cages (n = 2/group), and kept in a temperature-controlled room (22°C) with a 12-h dark-light cycle (light 07:00–19:00 h). The Medical School of the University of Sao Paulo Institutional Animal Care and Use Committee approved the experimental protocol, and this study was conducted in accordance with the National Institutes of Health (NIH) Guide for the Care and Use of Laboratory Animals. Rats were randomly assigned to 2 experimental groups: control (CG, n = 10) and diabetic (DG, n = 8).

### Experimental diabetes model

Experimental diabetes was induced by intravenous injection of 50 mg/kg of STZ (Sigma Chemical Co., St. Louis, MO) dissolved in citrate buffer (pH 4.2). Food was withheld from the rats for 6 hours before STZ injection. Control rats received a placebo (10 mM citrate buffer, pH: 4,5) after a similar fasting period. 48 hours after STZ injection, blood glucose levels above 200 mg/dL confirmed the diabetic state.

### Measurement of Oxygen Consumption and Maximal Exercise Test

Sixty days after STZ-induction of diabetes, the animals were placed on a treadmill and enclosed in an airtight metabolic chamber. This chamber was adapted for the determination of O_2 _uptake (VO_2_) by using an open-circuit method. After 30 minutes of rest on the treadmill, VO_2 _basal was collected. VO_2 _was measured by means of expired gas analysis during a ramp protocol of a progressive exercise test, which consists on a treadmill exercise with 3 m/min increments every 3 minutes, and finishes when VO_2 _max is reached. VO_2 _max was defined as the VO_2 _after which an increase in work rate was not associated with a further increase (± 5%) in continuously measured O_2 _uptake. The metabolic chamber-enclosed treadmill was airtight, except for the front inflow and rear outflow port. Appropriate inspired PO_2 _was delivered from gas thanks with known concentrations. Inflow was maintained constant at ~6 l/min. Inflowing and outflowing O_2 _concentrations were measured continuously through an oxygen analyzer (S-3A/I, Ametek, Pittsburgh, PA, USA).

VO_2 _was calculated using the measured flow through the metabolic chamber (PF), the expired fraction of effluent oxygen (FEO_2_), the fraction of oxygen in room air (FIO_2_), and animals body weight (BW), by the formula previously described by Rolim et al [17]:

VO_2 _(mL/Kg^-1^/min^-1^) = PF * (FIO_2 _- FEO_2_)/BW

The VO_2 _reserve was calculated by the formula:

VO_2 _res (mL/Kg^-1^/min^-1^) = VO_2 _maximum - VO_2 _basal

### Statistical analysis

The data are reported as mean ± SEM. A Pearson's product-moment correlation coefficient and univariate was used to evaluate the relationship between: (a) ET and VO_2_; (b) ET and VO_2 _res; (c) VO_2 _measured and VO_2 _predicted by equation, and (d) VO_2 _res measured and VO_2 _res predicted by equation. ANOVA one way, followed by the Student-Newman-Keuls post-hoc test was used to compare groups. Statistical significance was established at p < 0.05. Data are reported as means ± SEM.

## Results

### Animal characteristics

The body weight was lower in DG (235 ± 13 g) than in CG (445 ± 16 g) (p < 0.05). The diabetic animals developed severe hyperglycemia (DG: 397 ± 9 mg/dL) compared with control animals (CG: 88 ± 4 mg/dL) (p < 0.05).

### Exercise capacity

Diabetic group presented reduced values of maximum running speed in ET compared with that observed in CG (19 ± 1 vs. 24 ± 1 m/min, p < 0.01). VO_2 _max (72.5 ± 1 vs. 81.1 ± 1 mL/Kg/min^-1 ^in CG, p < 0.01), and VO_2 _res max (34 ± 2 vs. 51 ± 1 mL/Kg/min^-1^in CG, p < 0.01) were also reduced in DG compared with that in CG.

### Relationship between ET speed and VO_2 _or VO_2 _reserve

**Figure 1:**
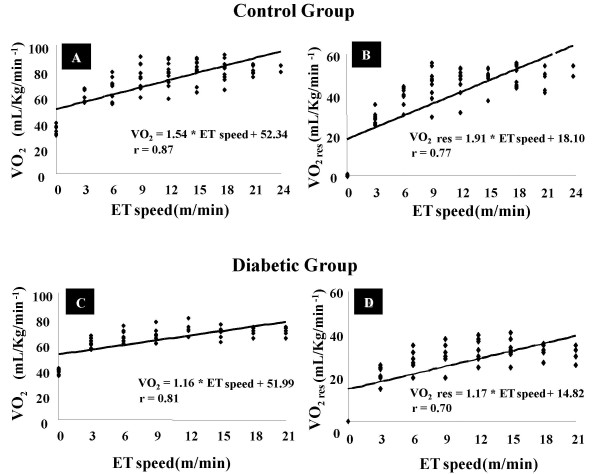
Relationships between VO_2 _and VO_2 _reserve (VO_2 _res) and exercise test speed (ET speed) in control (CG) and diabetic (DG) groups.

VO_2 _of both studied groups presented a linear increase during incremental speed protocols. In fact, we observed positive correlations between ET speed and VO_2 _in CG (r = 0.87; p < 0.05) (Figure 1A) and DG (r = 0.81; p < 0.05) (Figure 1C). Moreover, additional positive correlations were observed between ET speed and VO_2 _reserve in both CG (r = 0.77; p < 0.05) (Figure 1B) and DG (0.70; p < 0.05) (Figure 1D). Linear regression analysis were used to obtain the equations for correlations between ET speed and VO_2_: CG (VO_2 _= 1.54 * ET speed + 52.34) and DG (VO_2 _= 1.16 * ET speed + 51.99), and ET speed and VO_2 _reserve: CG (VO_2 _res = 1.91* ET speed + 18.10) and DG (VO_2 _res = 1.17* ET speed + 14.82).

We also observed a positive correlation between maximum speed of ET and VO_2 _max for both studied groups: CG (r = 0.7; p < 0.05) and DG (r = 0.7; p < 0.05). Thus, this data suggests that higher maximal speeds achieved on ET are associated with higher VO_2 _max values.

### Relationship between VO_2 _and VO_2 _reserve measured versus predicted

We tested the correlations between measured VO_2 _and VO_2 _predicted by linear regression equation to validate previously proposed predictive equations, and the same was done for VO_2 _reserve. There were positive correlations between VO_2 _measured and VO_2 _predicted (r = 0.81, p < 0.01 for CG; and r = 0.75, p < 0.05 for DG), and also between VO_2 _res measured and VO_2 _res predicted (r = 0.83, p < 0.01 for CG; and r = 0.75, p < 0.03 for DG) (Table 1).

**Table 1:** Relationships between measured VO_2 _and VO_2 _reserve with predicted VO_2 _and VO_2 _reserve.

	**Variables**	**Mean ± SEM (ml/Kg/min)**	**r Value**	**r^2 ^Value**	***p *Value (Pearson correlation)**	**SEE**
**CG**	**VO_2 _max measured**	81.1 ± 1	0.81	0.66	0.0077	9.7
	**VO_2 _max predicted**	89.5 ± 4				
	**VO_2 _res max measured**	51.2 ± 1	0.83	0.69	0.0053	9.4
	**VO_2 _res max predicted**	56.0 ± 5				

**DG**	**VO_2 _max measured**	72.5 ± 1*	0.75	0.56	0.0314	7.7
	**VO_2 _max predicted**	74.6 ± 3*				
	**VO_2 _res max measured**	34.4 ± 2*	0.75	0.57	0.0289	7.7
	**VO_2 _res max predicted**	32.2 ± 3*				

Data are reported as mean ± SEM. r and r^2 ^values, standard error estimate – SEE and *p *values of Pearson correlation are presented; VO_2 _max: maximal oxygen consumption; VO_2 _res max: reserve maximal oxygen consumption; CG: control group; DG: diabetic group; * p < 0.05 vs. CG (one-way ANOVA).

### Relationship between percentiles of ET and percentiles of VO_2 _and VO_2 _reserve

Exercise prescriptions are commonly based on velocities corresponding to 40, 60, and 85% of maximum speed of ET. Therefore, we calculated the speed values corresponding to these percentages, considering 24 ± 1 and 19 ± 1 m/min as 100% of ET for CG and DG, respectively.

**Figure 2:**
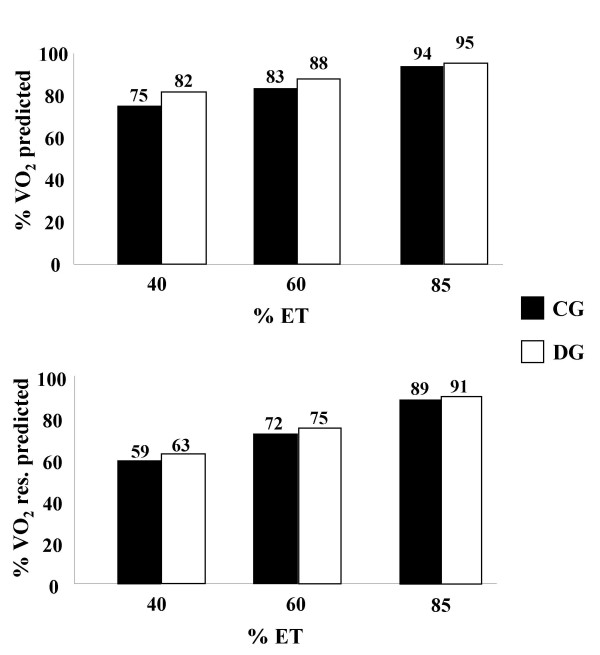
Corresponding percentiles of VO_2 _predicted and VO_2 _reserve predicted (VO_2 _res. predicted) based on 40, 60 and 85% of exercise test (%ET) in control (CG) and diabetic (DG) groups.

The speed (m/min) corresponded to each percentile value of ET was applied in the linear regression equations that correlate ET speed and VO_2_, absolute or reserve, to obtain the estimated VO_2_, as seen in Table 2. Finally, the percentiles were calculated for each value of VO_2 _and VO_2 _reserve obtained from 40, 60, and 85% of the maximum speed of ET. Corresponding percentiles of VO_2 _predicted and VO_2 _reserve predicted based on 40, 60 and 85% of ET are shown in Figure 2.

**Table 2:** Relationship between percentiles of exercise test and percentiles of VO_2 _and VO_2 _reserve.

	**%ET**	**ET speed predicted (m/min)**	**VO_2 _predicted (ml/Kg/min^-1^)**	**VO_2 _predicted**	**VO_2 _reserve predicted (ml/Kg/min^-1^)**	**%VO_2 _predicted reserve**
**CG**	**40**	10	67	75	34	59
	**60**	14	75	83	41	72
	**85**	20	84	94	51	89
	**100**	24	89	100	56	100

**DG**	**40**	8	61	82	23	63
	**60**	12	66	88	27	75
	**85**	17	71	95	33	91
	**100**	19	75	100	36	100

Values calculated by linear regression equation (Pearson's correlation coefficient and univariate) from percentiles (40, 60, 85 and 100%) of exercise test. ET: exercise test; VO_2_: oxygen consumption; CG: control group; DG: diabetic group.

## Discussion

The worldwide epidemic of diabetes that have emerged with the dawning of the 21st century has shown to be a major public heath problem, having struck developed countries as well as those still developing [1]. The diabetes induced by streptozotocin has been a largely used in animal models to study diabetic-induced cardiovascular and autonomic dysfunction [5,18-20].

It is well known that laboratory animals are constantly used in exercise physiology and cardiovascular rehabilitation research. However, the measurement of oxygen consumption in small animals is, many times, a limitation factor for research in this area, not only because of the high costs of gas analyses systems [7,21], but also because of two other important factors. First, the VO_2 _measurement must be carried out in a separate place from the rest of the laboratory to prevent alterations in the concentration of gases around the environment during data acquisition, and second, it is time-consuming because VO_2 _stabilization can take some time in rats. Moreover, the closed environment of the respiratory chamber may provoke one more stress situation for the experimental animal.

Although various instruments for human VO_2 _and VO_2 _reserve acquisition exist, to our knowledge, the respiratory chamber is the only one permitting continuous direct monitoring of VO_2 _in laboratory animals [17].

Studies of our laboratory have been conducted to investigate the benefits of exercise training in STZ-diabetic rats [6,14,16] and others pathological conditions [10,13,15], using the exercise training prescription based on maximal exercise test. In the present study, we have demonstrated that STZ-diabetes reduces VO_2 _max, VO_2 _res max and maximum running speed on ET. Furthermore, we also obtained significant correlations between VO_2 _and ET speed, VO_2 _reserve and ET speed and VO_2 _max and maximum ET speed in control and STZ-diabetic rats.

Accurate monitoring of VO_2 _consumption healthy or sick subjects is of great interest to medical research [22,23]. Researches have been wide for indirect forms of estimating VO_2 _max [7,24,25]. In 1954, Astrand & Ryhming [24] published a nomogram to predict VO_2 _max from submaximal pulse rates (120 to 170 bpm). Some years later, this nomogram was modified, and it is still being used today [26]. Balke & Ware [27], in 1959, developed a new protocol, and established a formula to calculate VO_2 _based on the speed and on treadmill slope [28]. In addition, recently, Maldonado-Martin et al. [29] demonstrated a good correlation between VO_2 _peak and the 6-minute walking test (6-MWT) in heart failure patients. However, these authors also concluded that when VO_2 _(peak) is predicted from equations using 6-MWT, the result is a substantial variability. Consequently, it should not be used in older HF patients, in which an accurate determination of functional capacity is essential. Currently, VO_2 _max, for both health men and women, can be estimated according to the formulas adopted by the American College of Sports Medicine [30].

The maximum VO_2 _values obtained in the present work are in accordance with previous findings for normal adult rats [31]. Likewise, the maximum speed values obtained in the exercise test are considered adequate values for rats and mice [7,13,15,26]. The reduction in VO_2 _max observed in diabetic rats can be due to many factors, such as cardiac output limitations [5,6], reduced peripheral blood flow [32] and problems in skeletal muscle metabolism [33].

The results of the present study provide values of VO_2 _for adult Wistar male rats at different treadmill speeds. In the range of treadmill speeds, 3 to 24 m/min for CG and 3 to 19 m/min for DG, VO_2 _increased progressively as a function of running speed, and it could be expressed by simple linear equations obtained by linear regression (VO_2 _= 1.54 * ET speed + 52.34, for CG; and VO_2 _= 1.16 * ET speed + 51.99, for DG). This equation allows VO_2 _estimation for one definitive workload, and it also gives the VO_2 _max from the maximum ET speed.

The increment in VO_2 _as a function of speed (Figure 1) was described in previous studies [34,35], and other predicted forms were proposed for Wistar-Kyoto and Spontaneously Hypertensive Rats [36]. However, our study has shown that STZ-diabetic rats present different slopes of VO_2 _in response to speed increment when compared to control rats. In fact, available data suggests that each experimental rat model needs a specific equation to predict the VO_2 _and VO_2 _reserve.

In this study, we tested the approximation between measured VO_2 _and VO_2 _reserve with VO_2 _and VO_2 _reserve predicted by linear regression equations in study groups. Significant correlations were observed between measured VO_2 _and VO_2 _reserve with predicted VO_2 _and VO_2 _reserve variables for CG (r = 0.81 and r = 0.83; p < 0.003) and DG (r = 0.75 and r = 0.75; p < 0.005), respectively. Thus, our study has showed that these equations, which are used to predict VO_2 _and VO_2 _reserve from ET, are valid for control and STZ-diabetic rats.

In exercise physiology, it is common and appropriate to characterize work intensity as percentage of VO_2 _max. In this study, it was observed that 60% of the maximum ET speed (CG: 14 m/min and DG: 12 m/min) corresponded to 83% of VO_2 _max and 72% of VO_2 _reserve in CG and 88% of VO_2 _max and 75% of VO_2 _reserve in DG. In animal models, a correlation between ET speed and VO_2 _was also established for adult and old C57BL/6J mice. In this study, the authors found that the speed of 12 m/min (~50% of the maximum ET speed) produced a VO_2 _equivalent to 76% and 89% of the VO_2 _max in adult and old mice, respectively [7]. Furthermore, Wisloff et al. [31] have demonstrated a linear relationship between VO_2 _and HR in sedentary and trained rats according to the increment of ET speed. Nevertheless, these authors emphasized that the maximum HR is not reached along with VO_2 _max. Instead, it is reached in intensities above VO_2 _max. Moreover, this study indicates that values of 90% of the maximum HR correspond to ~80% of the VO_2 _max.

It is important to highlight the positive correlations observed in the present study between maximum ET speed and VO_2 _max in CG (r = 0.7, p < 0.05) and DG (r = 0.7, p < 0.05). These correlations demonstrate that rats with higher ET performance presented higher VO_2 _max. As a result, this finding suggests that a simple ET is capable of detecting differences in the cardio-respiratory capacity of control and diabetic-STZ rats, and it also suggests that the improvement in cardio-respiratory performance (VO_2_max) after a period of physical training could be detected by maximum ET. In this aspect, it is important to emphasize that Noakes et al. [37] previously demonstrated that the highest speed reached in maximum ET is a better predictor of performance in laboratory tests when compared to %VO_2 _max, VO_2_max or racing speed at an extreme lactate threshold among marathon athletes. In our group, we have used ET as an indicator of improvement of exercise capacity after exercise training in diabetic, hypertensive ovariectomized rats and in normal mice [12-16].

## Conclusion

The results of the present study demonstrated that VO_2 _can be estimated from the results of ET in control and STZ-diabetic rats. This estimate can be assessed by obtaining equation by linear regression between VO_2 _and ET. The correlation between ET speed and VO_2 _showed that physical training prescription based on ET is adequate for control and diabetic animals. Furthermore, our data indicates that ET can detect differences in aerobic performance, as the maximum speed achieved in the ET was correlated with the maximum VO_2 _in these evaluated experimental models.

Therefore, we concluded that maximal exercise test can be used as an indicator of cardio-respiratory capacity, and it can be useful in further studies that investigate physiological effect of acute or chronic exercise on control and STZ-diabetic male rats.

## Abbreviations

VO_2_: oxygen consumption

VO_2 _max: maximum oxygen consumption

VO_2 _res: oxygen consumption of reserve

ET: exercise test

STZ: streptozotocin

CG: control group

DG: diabetic group

## Competing interests

The author(s) declare that they have no competing interests.

## Authors' contributions

The authors BR, DMF, CTM and MVH carried out the exercise test, maximal oxygen consumption data acquisition, performed the statistical analyses and discussion of results. BR, MCI and KDA participated in the study design and alignment, discussion of results, statistical analyses, and drafted the manuscript. MCI and KDA conceived the study, and participated in its design and coordination. All authors read and approved the final manuscript.
